# Stereotactically-guided craniotomy for resection of cerebral cavernous hemangioma: a series case report of 8 cases in Rasoul-e-Akram hospital (Tehran, Iran)

**Published:** 2012-11

**Authors:** Mansour Parvaresh

**Affiliations:** ^a^Department of Neurosurgery, Tehran University of Medical Sciences, Tehran, Iran.

## Abstract

**Background:**

In line with recent advances in magnetic resonance imaging (MRI), cavernous hemangioma (cavernoma) can be detected efficiently and reliably in patients with intractable epilepsy that surgical resection is a common treatment for them. Because these lesions are usually small and may be located near the eloquent areas, stereotactically-guided resection should be considered. This study aims to report the surgical features and outcomes of 6 patients underwent the stereotactically-guided craniotomy for excision of cavernoma.

**Methods:**

A total of six patients (1 male and 5 female, aged 10-42 years, mean age of 29 years) with cavernoma underwent stereotactically-guided craniotomy surgery in the Rasoul-e-Akram Hospital, affiliated to Tehran University of Medical Sciences (Tehran, Iran) for resection of cerebral cavernous hemangioma during September 2007 to May 2012. Clinical presentations were seizures, headache and hemorrhage. Two patients had a previous history of intracranial hemorrhage. Diagnosis was made using MRI and digital subtraction angiography. Locations of the lesions were parietal, frontal and temporoparietal. Size of lesion ranged from 0.7 to 3.1 cm (mean 1.7 cm). All those lesions were deep-seated or located near or within eloquent areas. Stereotactically-guided craniotomy was performed for excision of lesion. Clinical follow-up period ranged 5-48 months (mean 28 months). In all patients, complete surgical resection was achieved as demonstrated by postoperative image studies.

**Results:**

There was no associated morbidity or mortality. Histological diagnosis was consistent with cavernoma in all cases. Clinical follow-up revealed that all patients had complete recovery from preoperative symptoms and patients with seizures showed marked improvement.

**Conclusions:**

In conclusion, stereotactic guided craniotomy in surgery of eloquent or deep-seated cerebral cavernous malformations represents a very accurate and safe approach.

**Keywords:**

Stereotactically-guided craniotomy, Cavernous hemangioma, Magnetic resonance imaging


					Volume 4, Suppl. 1 Nov 2012
Publisher: Kermanshah University of Medical Sciences
URL: http://www.jivresearch.org
Copyright:
Congress of Iranian Neurosurgeons, 14-16 November 2012, Kermanshah, Iran
Paper No. 23
Stereotactically-guided craniotomy for resection of cerebral
cavernous hemangioma: a series case report of 8 cases in
Rasoul-e-Akram hospital (Tehran, Iran)
Mansour Parvaresh a,*
a
Department of Neurosurgery, Tehran University of Medical Sciences, Tehran, Iran.
Abstract:
Background: In line with recent advances in magnetic resonance imaging (MRI), cavernous hemangioma (cavernoma)
can be detected efficiently and reliably in patients with intractable epilepsy that surgical resection is a common
treatment for them. Because these lesions are usually small and may be located near the eloquent areas,
stereotactically-guided resection should be considered. This study aims to report the surgical features and outcomes
of 6 patients underwent the stereotactically-guided craniotomy for excision of cavernoma.
Methods: A total of six patients (1 male and 5 female, aged 10-42 years, mean age of 29 years) with cavernoma
underwent stereotactically-guided craniotomy surgery in the Rasoul-e-Akram Hospital, affiliated to Tehran University
of Medical Sciences (Tehran, Iran) for resection of cerebral cavernous hemangioma during September 2007 to May
2012. Clinical presentations were seizures, headache and hemorrhage. Two patients had a previous history of
intracranial hemorrhage. Diagnosis was made using MRI and digital subtraction angiography. Locations of the
lesions were parietal, frontal and temporoparietal. Size of lesion ranged from 0.7 to 3.1 cm (mean 1.7 cm). All those
lesions were deep-seated or located near or within eloquent areas. Stereotactically-guided craniotomy was
performed for excision of lesion. Clinical follow-up period ranged 5-48 months (mean 28 months). In all patients,
complete surgical resection was achieved as demonstrated by postoperative image studies.
Results: There was no associated morbidity or mortality. Histological diagnosis was consistent with cavernoma in all
cases. Clinical follow-up revealed that all patients had complete recovery from preoperative symptoms and patients
with seizures showed marked improvement.
Conclusions: In conclusion, stereotactic guided craniotomy in surgery of eloquent or deep-seated cerebral cavernous
malformations represents a very accurate and safe approach.
Keywords:
Stereotactically-guided craniotomy, Cavernous hemangioma, Magnetic resonance imaging
* Corresponding Author at:
Mansour Parvaresh: Department of Neurosurgery,Tehran University of Medical Sciences, Tehran, Iran, Tel:09121008574,
Email: m_parvaresh@yahoo.com, (Parvaresh M.).

				

